# Solving an emergency rescue materials problem under the joint reserves mode of government and framework agreement suppliers

**DOI:** 10.1371/journal.pone.0186747

**Published:** 2017-10-27

**Authors:** Jiang-Hua Zhang, Xiao-Qing Sun, Rui Zhu, Ming Li, Wang Miao

**Affiliations:** 1 School of Management, Shandong University, Jinan, Shandong, China; 2 School of Business Administration, Qilu University of Technology, Jinan, Shandong, China; Johns Hopkins University Bloomberg School of Public Health, UNITED STATES

## Abstract

Emergency rescue material reserves are vital for the success of emergency rescue activities. In this study, we consider a situation where a government owned distribution center and framework agreement suppliers jointly store emergency rescue materials. Using a scenario-based approach to represent demand uncertainty, we propose a comprehensive transportation pattern for the following supply chain: “suppliers—government distribution center—disaster area.” Using a joint reserves model that includes the government and framework agreement suppliers, we develop a non-linear mathematic model that determines the choices of the framework suppliers, the corresponding optimal commitment quantities, and the quantity of materials that are stored at a government distribution center. Finally, we use IBM ILOG CPLEX to solve the numerical examples to verify the effectiveness of the mode and perform sensitivity analyses on the relevant parameters.

## 1. Introduction

In recent years, numerous countries around the world have suffered from many natural disasters, which not only caused great loss of life and property but also affected a large number of individuals. For example, Super Typhoon Soudelor affected 1.584 million individuals, and 14 people died on August 2015 in China. The Nepal earthquake that occurred on April 25 of 2015 affected 300,000 individuals, and 140,000 of these were severely affected. In March of 2011, the East Japan earthquake affected 100,000 individuals and caused 20,000 deaths. According to EM-DAT (i.e., Emergency Events Database), 346 natural disasters occurred at home and abroad in 2015 and affected a total of 9.86 million people and resulted in 22,773 deaths. When disasters occur, the number of affected individuals increases rapidly within a short period of time, and the demand for emergency supplies sharply increases, which results in the need for material reserves that allow relief agencies to distribute emergency relief supplies in a short period of time.

Three primary modes are used to supply emergency rescue materials. For the first mode, rescue agencies (in China, this refers to the government) store relief supplies in pre-disaster facilities, package disaster relief supplies after a disaster occurs then directly transport these packages to disaster areas. For the second mode, a relief organization purchases emergency relief materials during the post-disaster period. However, post-disaster procurement may be an extremely time-consuming process because most organizations use a competitive bidding procedure, and the entire procurement process (i.e., generating and announcing appeals, waiting for supplier bids, and evaluating bids [1]) begins only after a disaster occurs. Therefore, this is not only a cumbersome process but also wastes precious time for rescues and involves significant risk. For the third supply mode, a framework supplier reserves emergency relief materials; to clarify, according to framework agreements, suppliers reserve protocol inventories for rescue organizations and agree to provide these supplies when they are needed. After a disaster, framework suppliers process the order and transport the supplies to the affected area. Although this supply mode can reduce pressures on the government’s financial and inventory resources, it cannot meet the material needs during the most urgent time period after a disaster occurs. Each of these three supply modes has shortcomings. Government reserves can meet needs immediately after a disaster, but this is a costly strategy and low liquidity and implies that large-scale government pre-disaster reserves cannot be achieved. Although a framework supplier can reduce pressures on government resources, it cannot meet all the needs of an emergency rescue, which implies that an emergency rescue effort cannot be undertaken solely by the supplier.

Based on the above discussion, this study focuses on a mode that consists of joint reserves held by the government and framework agreement suppliers. Prior to the disaster, the government itself reserves a certain amount of emergency relief supplies. At the same time, the government needs to look for framework agreement suppliers and develops framework agreements with suppliers. Under the framework agreement, the supplier is committed to reserve a certain amount of emergency rescue supplies. After the disaster, the government first puts its own pre-disaster relief supplies into the disaster area to meet the most urgent needs. Then the framework suppliers transport their pre-disaster relief supplies to the government distribution center where relief supplies are packaged in proportion to human needs and transported to the disaster area. For this mode, the government and framework agreement suppliers store a certain amount of materials to ensure that pre-disaster reserves can be transported to a disaster site to meet the most urgent needs in the shortest possible time, and the government and suppliers continue to supply follow-up relief supplies that can be transported to the affected area. Therefore, the reserve costs and supply costs of the rescue agencies’ emergency relief materials are kept at low levels. The primary focus of this study is to consider a joint reserves mode that includes government and framework suppliers. This study also focuses on methods to better coordinate the government's pre-disaster reserves and the framework suppliers’ reserves and determine the optimal reserve volume. We develop an optimal joint reserves model that determines the framework suppliers and the corresponding optimal commitment quantity in addition to determining the pre-disaster amount of reserves that are stored in the government's emergency material warehouse.

This paper includes five sections. In Section 1, we introduce the research background and the framework of this study. In Section 2, we briefly summarize related studies. In Section 3, we define the problem and build a mathematical model to select the framework agreement suppliers, determine the material commitment quantities and determine the storage amount that the rescue agencies need for pre-disaster materials. In Section 4, we validate the model using a numerical example and conduct a sensitivity analysis of the relevant parameters to determine how the reserves and supply costs of the emergency rescue materials change for different conditions. In Section 5, we conclude this paper by briefly summarizing this study and highlighting possible areas for future study.

## 2. Literature review

Numerous studies have been conducted regarding emergency relief material reserves. These studies can be categorized into two problems: the first problem is to study the entire network of the emergency rescue from a macro perspective, which primarily includes the location of the material reserve points and a determination regarding the storage quantity of materials needed during the emergency rescue process. The second problem is to analyze the material reserve decisions for each supply point from a micro perspective, including decisions regarding safety stocks, order cycles and order quantities.

### 2.1 Macroscopic research

The macro aspects of material reserves include problems related to infrastructure allocations of emergency relief work. These aspects also include the locations of evacuation facilities, distribution centers, and inventory facilities and site selections of medical facilities. One problem related to the locations of material storage facilities is determining the corresponding reserve points for each material demand point. After a disaster occurs, the process must ensure that the demand points can receive a timely rescue and the needed supplies.

Toregas [2] uses the ensemble coverage theory to study the site selection of emergency facilities. A linear programming model constructed by Toregas is one of the earlier studies regarding facility locations in emergency areas. Balcik and Beamon [3] use the classical maximum coverage model for this location problem and establish a location model for a distribution center involved in the rescue process. This model provides an effective solution for the organization of pre-disaster facilities and material deployment, including location decisions and the corresponding material reserves for the rescue distribution center (RDC) location. Ukkusuri and Yushimito [4] construct a site-path model that includes the location and inventory of material storage facilities and the distribution route of post-disaster materials, which also considers possible damage of post-disaster roads and introduces the concept of reliable routes. In addition, this model is used to conduct sample tests for the famous Sioux Falls network. Mohammadi et al. [5] propose a multi-objective, mixed-integer programming model to maximize the satisfaction of post-disaster demand, the lowest cost of material reserves and the optimal fairness in the distribution of post-disaster materials. Dekle [6] et al. perform a study on the location of the Florida Disaster Recovery Center (DRC). These scholars use the standard ensemble coverage model to select the optimal site for the DRC at a distance of 20 miles, which satisfies other evaluation conditions by relaxing conditions based on a pre-determined optimal site-location. Jing Wang et al. [7] establish a two-stage stochastic programming model to solve the division of material reserve areas. In the following empirical study, it is determined that resources divided into the same area can be shared. Campbell and Jones [8] construct a formula to determine optimal material reserves, minimize the total expected cost from the supply point to the demand point, and determine the optimal supply point location by using a cost model. Widener [9] classifies the material distribution facilities (POD) that respond to hurricane hazards into three grades according to the type of materials they provide. This scholar assumes that high-grade facilities can provide materials that are provided by low-level facilities and assumes that low-grade facilities and high-grade facilities provide basic and special materials, respectively. He optimizes the median location model with capacity constraints, combines the relevant geographic information technology, and finally establishes a spatial optimization model for the hierarchical distribution of location centers. Lang and McGarvey [10] identify a reliable network posture, which is a set of utilized facility locations and an allocation of materiel to those locations, that can satisfy time-sensitive delivery requirements to potential locations around the globe, ensuring that demands can be satisfied even in the event of loss of access to a subset of storage sites, all at minimum total cost. And they develop new optimization formulations to account for differing levels of network reliability.

### 2.2 Microscopic research

In studies regarding relief supplies inventories, certain scholars analyze the cost problem of multiple modes and consider issues related to emergency stocks and transportation [11–13]. Ozbay and Ozguven [14] build a time-based inventory control model that ensures an effective supply of materials to be distributed during the disaster recovery process. Ozguven and Ozbay [15] also introduce a multi-commodity stochastic inventory control model by introducing Radio Frequency Identification (RFID) technology into disaster relief operations to track the inventory in real time and determine the amount of material that is needed prior to and after the disaster. This model provides a solution for rescue organizations to maintain safe and effective stock levels at the lowest cost possible. In order to overcome the incomplete information and uncertain time, Qiu and Zhang et al. [16] establish the nonlinear programming model that objective function is the maximization of time-satisfaction degree to select the reliable and optimal path. Das and Hanaoka [17] focus on the lead time of the materials order and demand uncertainty as a continuous uniform distribution function in disaster relief. Based on this perspective, the order point problem of material reserves is analyzed and effectively avoids rescue losses that are caused by shortages in materials. Rawls and Turnquist [18] construct a two-stage random mixed-integer program (SMIP) that determines the amount of various types of pre-position emergency supplies and determines the uncertainty of the ultimate need. Considering a traffic jam that is caused by a possible evacuation and time constraints, Davis and Samanlioglu et al. [19] propose a stochastic programming model that determines how a cooperative warehouse network should allocate relief supplies and coordinate material stocks between warehouses. Sheu [20] proposes a hybrid fuzzy clustering optimization method to manage the emergency logistics distribution of relief supplies during an emergency rescue. Garrid, Lamas and Pino [21] build a model that optimizes stock levels of emergency supplies and vehicle availability to provide sufficient supply to meet demands with given probabilities. Taskin and Lodree [22] present a model that solves stochastic inventory for companies that produce and procure emergency relief supplies and are confronted with challenges related to the hurricane season. Beamon and Kotleba [23] consider an emergency inventory model for humanitarian organizations during complex emergency situations (generally long-term political disasters). To determine the characteristics of a complex emergency cycle and uncertain demand, Beamon and Kotleba build a stochastic inventory control model where the order point and order quantity can be determined by the order cost, the inventory cost and the repurchase cost. Perez-Rodriguez and Holguin-Veras [24] develop an inventory-allocation distribution model for post-disaster humanitarian logistics with explicit consideration of deprivation costs. This model minimizes social costs, designs suitable heuristic solution approaches, and assesses the performance of the heuristics using numerical experiments.

By analyzing these prior studies, we note that most scholars have engaged in considerable analyses regarding the locations of emergency material reserves, the framework agreement emergency material reserves model and the inventory of relief materials. However, most of these studies only consider the suppliers’ reserves or the rescue organization pre-disaster reserves. These studies do not consider combining the pre-disaster reserves of the rescue organization and the reserves of the framework agreement supplier, which may drastically increase the efficiency of emergency material reserves and supplies. Furthermore, few scholars conducted quantitative analyses.

Balcik and Ak [25] consider that a rescue organization signs an emergency relief material reserves framework agreement with a supplier, which stipulates that after the disaster, the supplier should deliver the purchased emergency relief goods directly to the affected area. If the procurement volume is less than the amount of the framework agreement, the rescue organization must pay compensation costs for the unneeded supplies. These scholars construct a dynamic programming model and illustrate the effectiveness of this model using a numerical example. However, this model includes shortcomings. First, Balcik and Ak specify the method used by the framework supplier to store the goods for the government; therefore, this approach does not meet the needs of the post-disaster emergency time period because it ignores the importance of government pre-disaster reserves. Second, suppliers directly deliver the purchased relief materials to the disaster area, which is likely to cause further damage to already damaged traffic conditions and may adversely affect important emergency rescue operations. To overcome these shortcomings, this study proposes the following methods. First, this study considers that the government and the framework suppliers jointly store the emergency rescue materials and constructs a model that determines the amount of pre-disaster reserves and the selection of the framework suppliers and their reserves. Second, this study proposes "suppliers—government distribution center—disaster area" mode of transport, which transfers the transport pressure to the distribution center (i.e., the government owned emergency material reserves) to significantly alleviate traffic pressure in the affected areas. Furthermore, according to scientific proportion, the distribution center distributes the different emergency relief supplies into numerous areas.

## 3. Problem and model

### 3.1 Problem description

In this study, the rescue agencies simultaneously have three functions: determining the amounts of framework agreement emergency relief supplies, post-disaster procurement, and pre-disaster reserves. These functions ensure that the government and enterprises coordinate the material reserves. For the government-enterprise joint scenario, the rescue efforts related to the emergency relief supply reserves can be divided into two stages: pre-disaster and post-disaster. Prior to the disaster, the rescue agencies select the appropriate suppliers for the framework agreement and determine a specific amount of storage for the framework agreement suppliers. Concurrently, the rescue agencies reserve a certain amount of materials to respond to the first post-disaster material needs in the distribution center, which can also act as the government owned emergency material repository. After the disaster occurs, the emergency relief supplies reserved by the distribution center are first used for the emergency rescue operations. Then, emergency relief supplies are continuously transported from the framework agreement providers to the distribution center; then, they are immediately packed and distributed to the disaster site.

**Fig 1:**
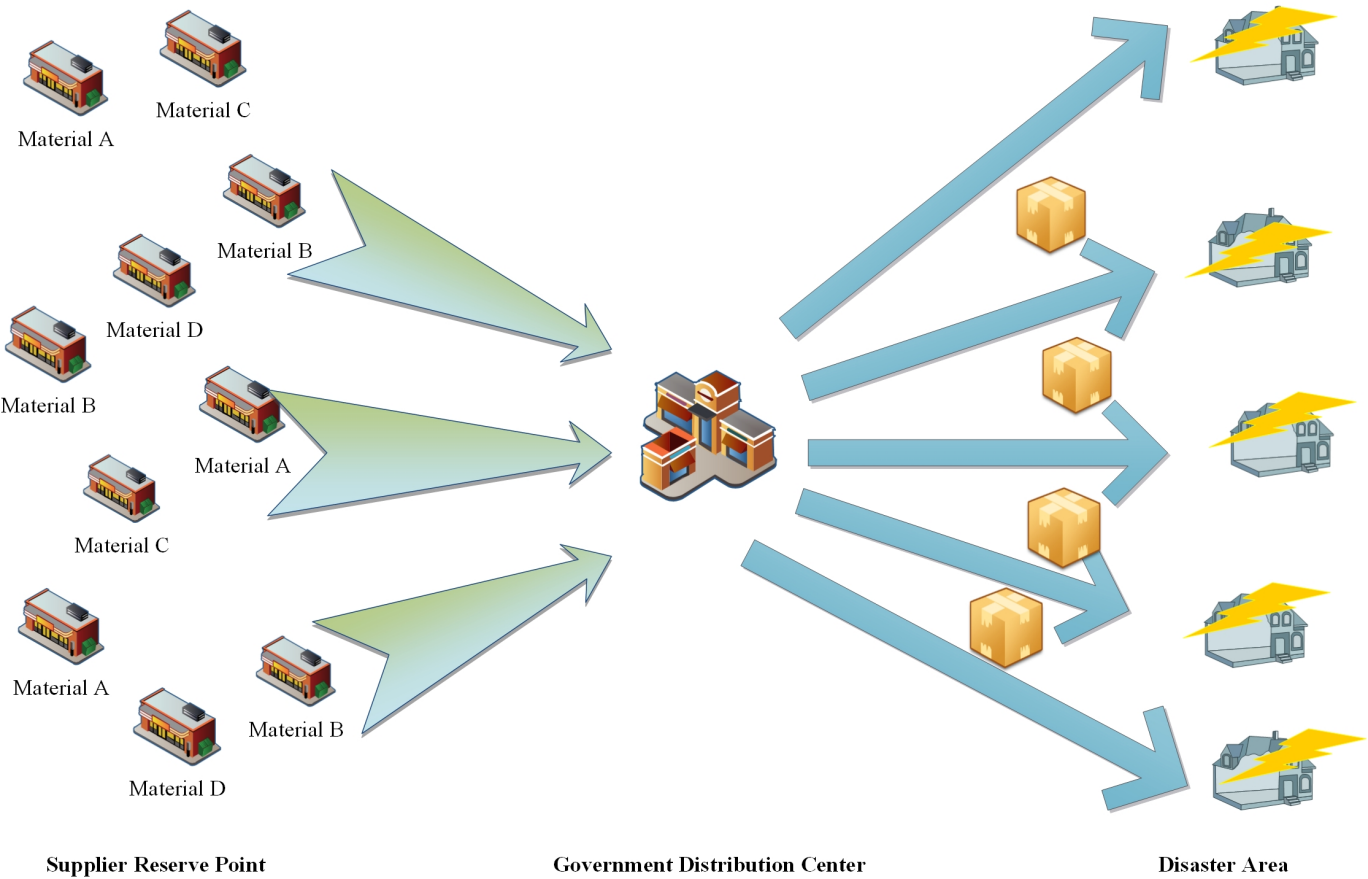
Rescue materials flow chart for the government-enterprises joint reserves mode.

After the disaster occurs, the transportation mode that is used by the supplier to transfer the emergency rescue materials to the disaster site is replaced by the transportation mode of "supplier—government distribution center—disaster area". The "government distribution center" refers to the "emergency rescue supplies pre-disaster reserves point". This mode intensifies the cooperation between the framework agreement supplier and the government from a transportation perspective. In regards to the transportation mode of “supplier—government distribution center—disaster area”, the supplier will deliver the emergency relief materials to the government distribution center according to the requirements of the rescue organization. In the government distribution center, relief agencies will pack all types of emergency materials into human needs packages and deliver these packages to the affected site. Fig 1 illustrates the flow of relief supplies for the government-enterprise joint reserves mode.

### 3.2 Model assumptions and symbol descriptions

#### 3.2.1 Model assumptions

1. Consider that two sources provide emergency relief supplies. One source is a supplier that is the framework agreement provider and the other source for supplies is the pre-disaster reserves from the government emergency material reserves. Because of the urgent need for emergency relief supplies, the framework agreement provider’s reserves are not sufficient to meet the entire demand. Because of the cost of the reserves, the emergency rescue supplies cannot be stored entirely at the government owned emergency reserve center. This study focuses on a combined effort of the framework agreement reserves and the government owned emergency reserves.
2. In this study, the rescue agencies, the government owned emergency material repository and the distribution centers are all owned by the government. In China, generally, rescue agencies are government agencies and the government also builds emergency material reserve centers around the country. The distribution center in this study refers to a local government emergency material reserve center.
3. This study only considers rapid consumables, such as water, food and medicine. The demand for this type of consumables, particularly water, medicines and food, is urgent and substantial at the disaster site.
4. This study assumes that the quantity of different materials have a fixed ratio. To clarify, the proportion of the mineral water, food, and medicine that is purchased by the relief organization from the supplier (each supplier can only provide one kind of material), the proportion of mineral water, food, drugs stored by the distribution center, and the packaging ratio of the distribution center are all consistent with a fixed proportion.

#### 3.2.2 Symbol description

Sets:

*N*: the set of candidate suppliers, *i* ∈ *N*;
*S*: the set of disaster scenario, *s* ∈ *S*; and
*K*: the set of emergency relief materials, *k* ∈ *K*.

Parameters:

*P*_*s*_: the probability of scenario *s*, *s* ∈ *S*;
*F*_*i*_: the selected cost of supplier *i*;
*f*_*k*_: the proportion of the *k-th* types of emergency relief supplies in the package;
*R*_*ik*_: the maximum reserve capacity of the framework agreement supplier *i* for emergency relief supplies *k*;
$d_{k}^{s}$: the demand of emergency relief supplies *k* in scenario *s*;
*g*_*k*_: the market prices of emergency relief supplies *k* that are procured from suppliers after the disaster occurs;
$m_{ik}=\left\{ \begin{array}{l} 1,\;\mathrm{If}\;\mathrm{supplier}\;i\;\mathrm{provides}\;\mathrm{material}\;k\\ 0,\;\mathrm{or}\;\mathrm{else} \end{array}\right.$;
*w*_*k*_: the unit compensation cost when the actual purchase volume of the rescue organization is less than the commitment quantity of the framework agreement suppliers;
*h*_*k*_: the supplier's storage cost of unit emergency supplies *k*;
$t_{ik}^{s}$: for scenario *s*, the unit transportation costs of the emergency relief material *k* from supplier *i* to the distribution center;
*j*_*k*_: the framework agreement price of one unit of emergency relief material *k*;
*c*_*k*_: the cost of purchasing and storing one unit of emergency relief material *k* at the distribution center;
*v*_*k*_: the minimum amount of emergency relief *k* stored at the distribution center;
*a*_*k*_: the minimum number of framework agreements suppliers that can provide the emergency relief material *k*;
*rmin*_*i*_: the lower limit of the framework agreement supplier *i* reserves account for its maximum reserve capacity; and
*rmax*_*i*_: the upper limit of the framework agreement supplier *i* reserves account for its maximum reserve capacity;
*M*: the auxiliary parameter for limiting certain variables.

Decision variables:

$Y_{i}=\left\{ \begin{array}{l} 1,\;\mathrm{If}\;\mathrm{supplier}\;i\;\mathrm{is}\;\mathrm{selected}\;\mathrm{as}\;\mathrm{the}\;\mathrm{framework}\;\mathrm{agreement}\;\mathrm{supplier}\\ 0,\;\mathrm{or}\;\mathrm{else} \end{array}\right.$;
*x*_*ik*_: the commitment amount of the emergency relief supplies *k* that is stored by supplier *i* as specified in the framework agreement;
*y*_*k*_: the storage capacity of the distribution center for the material *k*; and
$z_{ik}^{s}$: for scenario *s*, the quantity of the emergency relief material *k* that is purchased by the distribution center (rescue organization) from supplier *i*.

### 3.3 Model construction

The total cost of the government-enterprise joint effort includes the pre-disaster costs and the post-disaster costs. Pre-disaster costs include the cost for selecting the framework agreement suppliers, the supplier’s storage cost of the materials, and the distribution center’s storage cost. Post-disaster costs include the costs of procuring materials from suppliers and delivering them to distribution centers after a disaster has occurred (i.e., the material purchasing cost and transportation cost). After the disaster, the quantity purchased by relief agencies from suppliers may be less than or greater than the volume that is specified in the framework agreement. Orders that are less than the commitment quantity of the framework suppliers must be paid for compensation costs and orders that exceed the commitment quantity of framework agreement suppliers must be purchased according to market prices.

The emergency relief supply reserves model for a government-enterprise joint effort is as follows.

$$\left\{ \begin{array}{l} \min\{\sum_{i\in N}F_{i}Y_{i}+\sum_{i\in N}\sum_{k\in K}h_{k}x_{ik}+\sum_{k\in K}c_{k}y_{k}+\sum_{s\in S}\sum_{i\in N}\sum_{k\in K}P_{s}[(x_{ik}-z_{ik}^{s})w_{k}+z_{ik}^{s}t_{ik}^{s}+z_{ik}^{s}j_{k}]\},\\ while\;x_{ik}\geq z_{ik}^{s};\\ \min\{\sum_{i\in N}F_{i}Y_{i}+\sum_{i\in N}\sum_{k\in K}h_{k}x_{ik}+\sum_{k\in K}c_{k}y_{k}+\sum_{s\in S}\sum_{i\in N}\sum_{k\in K}P_{s}[(z_{ik}-x_{ik})g_{k}+z_{ik}^{s}t_{ik}^{s}+x_{ik}j_{k}]\},\\ while\;x_{ik}\leq z_{ik}^{s}; \end{array}\right.$$ (1)

*s.t.*

$$f_{k}\sum_{k\in K}\left(\sum_{i\in N}z_{ik}^{s}+y_{k}\right)=\sum_{i\in N}z_{ik}^{s}+y_{k},\;\forall k\in K,s\in S$$ (2)

$$f_{k}\sum_{k\in K}y_{k}=y_{k},\;\forall k\in K$$ (3)

$$\sum_{k\in K}m_{ik}=1,\forall i\in N$$ (4)

$$\sum_{i\in N}z_{ik}^{s}+y_{k}\geq d_{k}^{s},\;\forall k\in K,s\in S$$ (5)

$$\sum_{i\in N}Y_{i}•m_{ik}\geq a_{k},\forall k\in K$$ (6)

$$\begin{array}{l} z_{ik}^{s}\leq (1-Y_{i}•m_{ik})M+R_{ik}\\ z_{ik}^{s}\leq M•Y_{i}•m_{ik},\;\forall s\in S,i\in N,k\in K \end{array}$$ (7)

$$x_{ik}\geq rmin_{i}•R_{ik}•Y_{i},\forall i\in N,k\in K$$ (8)

$$x_{ik}\leq rmax_{i}•R_{ik}•Y_{i},\forall i\in N,k\in K$$ (9)

$$y_{k}\geq v_{k},\forall k\in K$$ (10)

$$x_{ik}\geq 0,\forall i\in N,k\in K$$ (11)

$$z_{ik}^{s}\geq 0,\;\forall s\in S,\;i\in N,\;k\in K$$ (12)

$$Y_{i}\in \left\{0,1\right\},\forall i\in N$$ (13)

(1) is the objective function that minimizes the total cost of the material reserves for the joint effort of the government and the enterprises. Constraint (2) indicates that the total amount of the procurement from suppliers and the amount of relief supplies at the distribution center should meet the fixed proportion requirement. Constraint (3) indicates that the amount of various types of emergency relief materials at the distribution center meet the fixed proportion requirement. Constraint (4) implies that each supplier can only provide one type of material. Constraint (5) indicates that the supply of materials is greater than the demand; to clarify, the supply of materials that is procured from the framework agreement suppliers and stored by the distribution center is greater than the demand of the disaster area. Constraint (6) limits the number of framework agreement suppliers; to clarify, the number of suppliers cannot be less than a specified number to avoid inadequate supply caused by a supplier breaking the contract. Constraint (7) implies that if the distribution center purchases goods from supplier *i*, it must ensure that supplier *i* is selected as the framework agreement supplier, where *M* is a sufficiently large positive number. Considering the accuracy and validity of the results, we set the value of *M* to 10^10^ (i.e., *M* = 10^10^). Concurrently, the quantity of the material that is purchased by the relief organization from suppliers shall not exceed the amount that is stored by the supplier. Constraints (8) and (9) ensure that the amount of the material that is specified by the supplier’s agreement is between specified minimum and maximum limits. Constraint (10) defines the lower limit of the amount of material in the distribution center. Constraints (11) and (12) are non-negativity constraints. Constraint (13) defines the binary variables.

### 3.4 Model transformation

The original model is a piecewise function and is also a nonlinear function. If IBM ILOG CPLEX is used to solve this function directly, it will be difficult to calculate. To facilitate the calculation, this study introduces a "0–1 variable" $u_{ik}^{s}$, where $u_{ik}^{s}=\left\{ \begin{array}{l} 1,x_{ik}\geq q_{ik}^{s}\\ 0,x_{ik}<q_{ik}^{s} \end{array}\right.$ and the constraint is $\left\{ \begin{array}{l} x_{ik}-z_{ik}^{s}\geq (u_{ik}^{s}-1)M\\ x_{ik}-z_{ik}^{s}\leq u_{ik}^{s}M \end{array}\right.$ for which *M* is a large positive integer and *M* = 10^10^.

When $x_{ik}\geq z_{ik}^{s}$ for the equation $x_{ik}-z_{ik}^{s}\geq (u_{ik}^{s}-1)M$ and the left side of the equation is a non-negative number, then $u_{ik}^{s}$ is equal to 0 or 1; for the equation $x_{ik}-z_{ik}^{s}\leq u_{ik}^{s}M$ when the left is a non-negative number, to ensure that the equation is constant, $u_{ik}^{s}$ can only be 1. Therefore, when $x_{ik}\geq z_{ik}^{s}$, $u_{ik}^{s}$ is 1.

When $x_{ik}<z_{ik}^{s}$ for $x_{ik}-z_{ik}^{s}\geq (u_{ik}^{s}-1)M$ and the left side of the equation is negative, to ensure that the equation is constant, the right can only be negative, and $u_{ik}^{s}$ is equal to 0; for the equation $x_{ik}-z_{ik}^{s}\leq u_{ik}^{s}M$, if the left side of the equation is negative, then $u_{ik}^{s}$ is either 1 or 0. Therefore, when $x_{ik}<z_{ik}^{s}$, $u_{ik}^{s}$ is 0.

The original model can be transformed into the following linear model:

$$\begin{array}{l} \min\{\sum_{i\in N}F_{i}Y_{i}+\sum_{i\in N}\sum_{k\in K}h_{k}x_{ik}+\sum_{k\in K}c_{k}y_{k}+\sum_{s\in S}\sum_{i\in N}\sum_{k\in K}u_{ik}^{s}P_{s}[(x_{ik}-z_{ik}^{s})w_{k}+z_{ik}^{s}t_{ik}^{s}+z_{ik}^{s}j_{k}]\\ +\sum_{s\in S}\sum_{i\in N}\sum_{k\in K}(1-u_{ik}^{s})P_{s}[(z_{ik}-x_{ik})g_{k}+z_{ik}^{s}t_{ik}^{s}+x_{ik}j_{k}]\} \end{array}.$$

When $x_{ik}\geq z_{ik}^{s}$, $u_{ik}^{s}$ is 1. The model is calculated as follows:

$$\min\{\sum_{i\in N}F_{i}Y_{i}+\sum_{i\in N}\sum_{k\in K}h_{k}x_{ik}+\sum_{k\in K}c_{k}y_{k}+\sum_{s\in S}\sum_{i\in N}\sum_{k\in K}P_{s}[(x_{ik}-z_{ik}^{s})w_{k}+z_{ik}^{s}t_{ik}^{s}+z_{ik}^{s}j_{k}]\}.$$

When $x_{ik}<z_{ik}^{s}$, $u_{ik}^{s}$ is 0. The model is calculated as follows:

$$\min\{\sum_{i\in N}F_{i}Y_{i}+\sum_{i\in N}\sum_{k\in K}h_{k}x_{ik}+\sum_{k\in K}c_{k}y_{k}++\sum_{s\in S}\sum_{i\in N}\sum_{k\in K}P_{s}[(z_{ik}-x_{ik})g_{k}+z_{ik}^{s}t_{ik}^{s}+x_{ik}j_{k}]\}.$$

After this transformation, the model is transformed into a linear model that can be calculated by IBM ILOG CPLEX. The linear model has the same results as the original model; therefore, the latter part of this paper uses the above model for calculations.

## 4. Case analyses

### 4.1 Problem description and parameter settings

In this section, we use an earthquake-prone province as an example and use relevant data to verify the validity of the model. The parameters in the model are set as follows.

1. Based on the immense amount of data collected from previous disasters (such as earthquakes), the probability of occurrence for each earthquake magnitude is calculated, which denotes the probability of the applied scenario. In this study, the level of possible earthquake disasters in the region is divided into four grades, so there are four kinds of disaster scenario and the set of disaster scenario is *S* = [1,2,3,4]. The probability ratio of the four levels of earthquake occurrence is assumed to be 4:3:2:1. The probability of a disaster occurrence is assumed to be 0.4; therefore, the probability of occurrence for each disaster scenario is *P*_*s*_ = [0.16,0.12,0.08,0.04].
2. Suppose there are 12 candidate suppliers (i.e., the set of candidate suppliers is *N* = [1,2,3, 4,5,6,7,8,9,10,11,12]) and each supplier provides various types, brands, and quality of materials; therefore, the agreement cost between the relief agency and each supplier differs and includes the fixed agreement cost and the holding cost subsidies for the agreement materials. The selection cost of the *i* th supplier is *F*_*i*_ = [230000,260000,270000,260000,290000,270000,280000,260000, 280000,280000,310000,300000], the unit is one yuan.
3. In Section 3.2, the paper assumes that we only consider rapid consumables. The affected population has more urgent demand for water, food and medicine in the disaster scenario. Therefore, we consider three kinds of emergency supplies, namely, mineral water, biscuits and medicines in this case. Therefore, the set of emergency relief materials is *K* = [1,2,3].To facilitate the packaging at the distribution center (i.e., government self- built relief material repository) and to distribute these packages to the affected areas, we assume that the quantity ratio of the three materials is 1:1:0.3 or *f* = [1,1,0.3].
4. $d_{k}^{s}$ refers to the demand of the various goods and materials by the affected areas considering various scenarios. The demand is highly related to the intensity of the disaster (i.e., disaster scenarios) and the disaster area. In general, as disaster intensity increases, more individuals are affected, and there is a greater demand for materials; as the disaster intensity decreases, the material demand also decreases. This study assumes that $d_{s}^{k}$ = [[300 300 90],[540 540 162],[1050 1050 315],[1500 1500 450]], and the unit is one thousand.
5. *m*_*ik*_ indicates whether supplier *i* provides material *k*; 1 indicates that material *k* can be provided and 0 indicates that the material *k* cannot be provided. We assume that suppliers 1, 2, 3, or 4 can provide the first type of emergency material, suppliers 5, 6, 7, or 8 can provide the second type of emergency material, and suppliers 9, 10, 11, or 12 can provide the third type of emergency material. Accordingly, the types of emergency materials that can be provided by the 12 suppliers are expressed in the following form: *m*_*ik*_ = [[1 0 0], [1 0 0], [1 0 0], [1 0 0],[0 1 0], [0 1 0], [0 1 0], [0 1 0],[0 0 1], [0 0 1], [0 0 1], [0 0 1]].
6. *R*_*ik*_ represents the maximum reserve capacity for emergency supplies *k* at supplier *i*, and the value is inferred from a supplier's material reserve capacity over a period of time. The data form is consistent with *m*_*ik*_; to clarify, if a supplier cannot provide relief material *k*, the amount of emergency relief material is zero. We assume that *R*_*ik*_ = [[300 0 0],[600 0 0],[400 0 0],[800 0 0],[0 500 0],[0 600 0],[0 450 0],[0 350 0],[0 0 200],[0 0 130],[0 0 400],[0 0 100]], and the unit is one thousand. In the framework agreement, there are minimum reserves for each supplier. The minimum amount is 5% of the maximum reserve capacity of the supplier, which is denoted by *rmin*_*i*_ = [0.05, 0.05, 0.05, 0.05, 0.05, 0.05, 0.05, 0.05, 0.05, 0.05, 0.05, 0.05]. Similarly, a maximum limit for the reserves is set for each supplier. The maximum value is a percentage of the maximum reserve capacity of the supplier, which varies due to the differences between the various suppliers and is represented by *rmax*_*i*_ = [0.5, 0.5, 0.5,0.5,0.5,0.6,0.3,0.5,0.5, 0.6,0.6,0.5].
7. This study assumes that the minimum limit of the three types of material reserves at the distribution center is 10000, 10000 and 3000, otherwise denoted by *v*_*k*_ = [10,10,3], and the unit is one thousand.
8. *h*_*k*_ represents the supplier's storage costs for the three types of emergency relief materials, and we assume that *h*_*k*_ = [80,160,500], and the unit is one yuan. For example, *h*_*1*_ = 80 means that the storage cost of every 1000 bottles of mineral water is 80 yuan, *h*_*2*_ = 160 indicates that the storage cost of every 1000 packets of biscuits is 160 yuan, and *h*_*3*_ = 500 implies that the storage cost of 1000 boxes of medications is 500 yuan.
9. We assume that the framework price of the three types of materials is *j*_*k*_, and the unit is one yuan. For example, *j*_*k*_ = [800,1600,5000] implies that every 1000 bottles of mineral water costs 800 yuan, every 1000 packets of biscuits costs 1600 yuan and it costs 5000 yuan for 1000 boxes of medications. Note that the medication is a mixture of various emergency medicines and does not refer to one specific medication. The unit compensation price is 0.1 times the framework price, that is *w*_*k*_ = 0.1 *j*_*k*_ = [800,1600,5000], and the unit is one yuan.
10. Assuming that the market price of the various materials after the disaster is *z*_*k*_ = [1600,3200,10000], and the unit is one yuan. The market price here refers to the market price of purchasing 1000 units of materials.
11. Suppose that the total cost of purchasing, storing and maintaining each 1000 units of the materials in the distribution center prior to the disaster is *c*_*k*_ = [1800,3600,11250], and the unit is one yuan.
12. $t_{ik}^{s}$ represents the unit transport cost, which is calculated as $t_{ik}^{s}=l_{i}\cdot e_{s}\cdot b_{k}$. Suppose that the distance between the suppliers and the distribution center is, *l*_*i*_ = [120,80,90,50,120,80,110,110,110,110,70,80] and the unit is in kilometers. Assuming that the impact coefficient of the disaster is *e*_*s*_ = [1.1,1.2,1.3,1.5], the more severe the disaster is, the more serious the traffic damage and the higher the transportation costs. This study assumes that the unit distance freight for the 1000 units of three materials is *b*_*k*_ = [0.15,0.15,0.3], and the unit is one yuan/km.
13. To prevent a situation where a single supplier cannot meet the needs of the disaster area during unexpected situations, we set the minimum number of framework agreement suppliers for each material to *a*_*k*_ = 2.

### 4.2 Result analysis

Using IBM ILOG CPLEX software to solve the above model, the results are provided in Table 1 and Table 2.

**Table 1:** Selection results and commitment quantities for each framework agreement supplier.

Supplier selection Results (In brackets, 1 for selected and 0 for not selected)	Quantities of Emergency Material 1 (Thousands)	Quantities of Emergency Material 2 (Thousands)	Quantities of Emergency Material 3 (Thousands)
**Supplier 1 (1)**	15		
**Supplier 2 (1)**	115		
**Supplier 3 (0)**	0		
**Supplier 4 (1)**	400		
**Supplier 5 (1)**		35	
**Supplier 6 (1)**		360	
**Supplier 7 (1)**		135	
**Supplier 8 (0)**		0	
**Supplier 9 (1)**			10
**Supplier 10 (0)**			0
**Supplier 11 (1)**			149
**Supplier 12 (0)**			0

**Table 2:** Storage quantities of various emergency rescue materials at the distribution center.

Emergency rescue materials	Storage number (Thousands)
**Emergency rescue material 1**	10
**Emergency rescue material 2**	10
**Emergency rescue material 3**	3

The Table 1 indicates that suppliers 1, 2, 4, 5, 6, 7, 9, and 11 are selected as the framework suppliers. Among these suppliers, suppliers 1, 2, and 4 store the first type of emergency relief supplies and the material commitment quantities are 15,000, 115,000, and 400,000, respectively. Suppliers 5, 6, and 7 store the second type of emergency relief supplies, and the commitment quantities are 35,000, 360,000, and 135,000, respectively. Suppliers 9 and 11 store the third type of emergency relief supplies, and the commitment quantities are 10,000, and 149,000, respectively.

Table 2 indicates that the number of stored emergency rescue materials at the distribution center is 10,000, 10,000 and 3,000. This is the minimum quantity of distribution center materials that is stipulated by the model constraint because the storage cost of the emergency relief supplies is higher than the framework agreement reserve price, the compensation cost, and the post-disaster market price. However, the distribution center must maintain a certain amount of material to be able to meet the first-time material needs after the disaster occurs; therefore, the relief agency should store the emergency rescue materials at the distribution center according to the minimum standards.

### 4.3 Sensitivity analysis

To understand the impact of each factor on the rescue cost, the supplier's framework commitment quantity and the storage number of the government distribution center, this study considers the demand satisfaction rate, the compensation cost and the post-disaster procurement cost as independent variables to perform a single factor sensitivity analysis and analyze their effects on the results.

#### 4.3.1 Sensitivity analysis of the demand satisfaction rate

To analyze variances in the disaster rescue cost and the material storage quantity for different demand satisfaction rates, this study conducts a sensitivity analysis on various material demand satisfaction rates. For different material demand satisfaction rates (i.e., a certain percentage of total demand $d_{k}^{s}$), we analyze changes in the rescue costs, the supplier’s framework commitment quantity, distribution center storage capacity and the post-disaster procurement cost.

**Fig 2:**
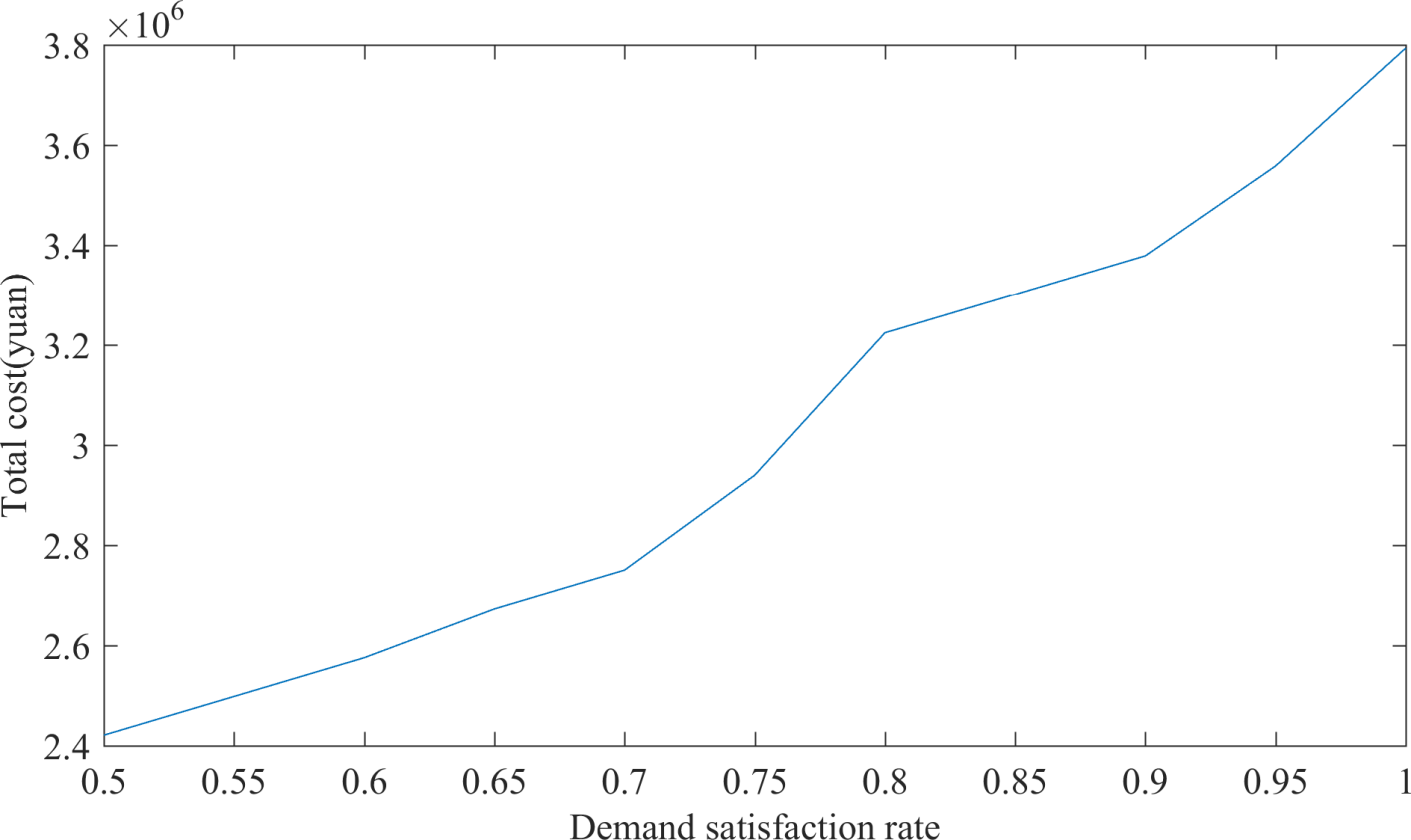
Trend of total cost as the demand satisfaction rate changes.

In this study, we analyze the total cost and changes in material reserves that correspond to 100% ~ 50% material demand satisfaction rates. The results are provided in Table 3. The relationship between the demand satisfaction rate and the total cost is illustrated in Fig 2.

**Table 3:** Total cost and material reserves for different demand satisfaction rates.

Demand Satisfaction Rate	Total cost (yuan)	Amount of storage at the distribution center (in thousands)	Supplier Selection and Protocol Reserves (in thousands)									
			1	2	4	5	6	7	8	9	10	11
**100%**	3793703.22	[10,10,3]	15	115	400	35	360	135	0	10	0	149
**95%**	3558378.59	[25,25,7.5]	0	88	400	0	360	110.5	17.5	10	0	136.4
**90%**	3378874.56	[10,10,3]	0	76	400	0	360	22.5	93.5	0	6.5	136.3
**85%**	3301529.43	[10,10,3]	0	49	400	0	360	22.5	665.	10	0	124.7
**80%**	3224266.33	[10,10,3]	0	30	392	0	230	22.5	169.5	0	6.5	120.1
**75%**	2939880.25	[25,25,7.5]	15	0	365	25	355	0	0	100	14	0
**70%**	2750286.23	[10,10,3]	15	0	353	0	345.5	22.5	0	100	10.4	0
**65%**	2672946.26	[10,10,3]	15	0	326	0	318.5	22.5	0	55.5	46.8	0
**60%**	2575548.21	[10,10,3]	15	0	299	0	296.5	0	17.5	87.7	6.5	0
**55%**	2498206.78	[10,10,3]	15	0	272	0	269.5	0	17.5	79.6	6.5	0
**50%**	2420913.78	[10,10,3]	15	0	245	0	242.5	0	17.5	71.5	6.5	0

Table 3 and Fig 2 indicate that when the demand satisfaction rate is between 50% and 70%, the total cost increases as the demand satisfaction rate increases and there is a linear relationship between these two variables. When the demand satisfaction rate increases from 70% to 80%, the growth trend of the total cost suddenly accelerates, which is significantly higher than the previous growth rate. In regards to the number of framework agreement suppliers, when the demand satisfaction rate is between 50% and 70%, there are six framework agreement suppliers. When the demand satisfaction rate reaches 80%, the number of framework agreement suppliers increases to seven. Because an increase in the number of framework agreement suppliers leads to an increase in the supplier selection cost, the total cost suddenly increases. Concurrently, the distribution center storage quantity also changes. When the demand satisfaction rate is between 50% and 70%, the distribution center storage quantity is [10,10,3], and when the demand satisfaction rate reaches 75%, the distribution center storage quantity rises to [25,25,7.5]. When the demand satisfaction rate reaches 80%, the distribution center storage quantities drop to [10,10,3] because the storage capacity of the six suppliers and the storage capacity of the distribution center (i.e., [10,10,3]) are sufficient to meet the demand when the demand satisfaction rate is between 50% and 70%. Therefore, the number of framework agreement suppliers and amount of storage at the distribution center do not change at this stage. When the demand satisfaction rate reaches 75%, the storage capacity of the six suppliers and the storage quantity of distribution center (i.e., [10, 10, 3]) cannot meet the needs, and the number of the framework suppliers must increase or the storage quantity at the distribution center to meet demand must increase. In this case, we increase the storage capacity of the distribution center and the number of framework agreement suppliers remains at 6, which is determined by a variety of costs. At this time, the cost of adding a framework agreement supplier is much larger than the material storage cost at the distribution center; therefore, the relief agency increases the quantity of materials stored at the distribution center rather than increasing the number of the framework agreement suppliers. When the demand satisfaction rate increases to 80%, the number of framework agreement suppliers increases from 6 to 7 and the material reserves at the distribution center decrease from [25,25,7.5] to [10,10,3]. As demand increases, if the number of framework agreement suppliers continues to remain unchanged, the distribution center can only increase the quantity of the material that is stored, and the material storage cost at the distribution center is high and uneconomical, which will lead to an increase in the number of framework agreement suppliers at a specific point to reduce the burden of the distribution center for storing supplies. When the demand satisfaction rate ranges from 80% to 95%, the growth trend of the total cost will return to prior levels. When the demand satisfaction rate reaches 100%, the total cost will sharply increase because the number of framework agreement suppliers increases from 7 to 8, which increases the selection costs. Concurrently, the storage capacity of the distribution center increases to [25,25,7.5] for a demand satisfaction rate of 95%; When the number of framework agreement suppliers is increased by one, the storage capacity of the distribution center falls again to [10,10,3] for the same reasons as previously described.

This result effectively indicates the coordination and flexibility of the emergency rescue material reserves model. In addition, the results demonstrate that for the government-enterprise joint reserves emergency rescue efforts, the material reserves of the framework agreement supplier is the primary source of relief supplies and the materials that are stored at the distribution center regulate this process. To ensure that the material demand can be met shortly after the disaster, the distribution center will maintain a certain quantity of stored materials.

#### 4.3.2 Sensitivity analysis of the unit compensation costs

**Fig 3:**
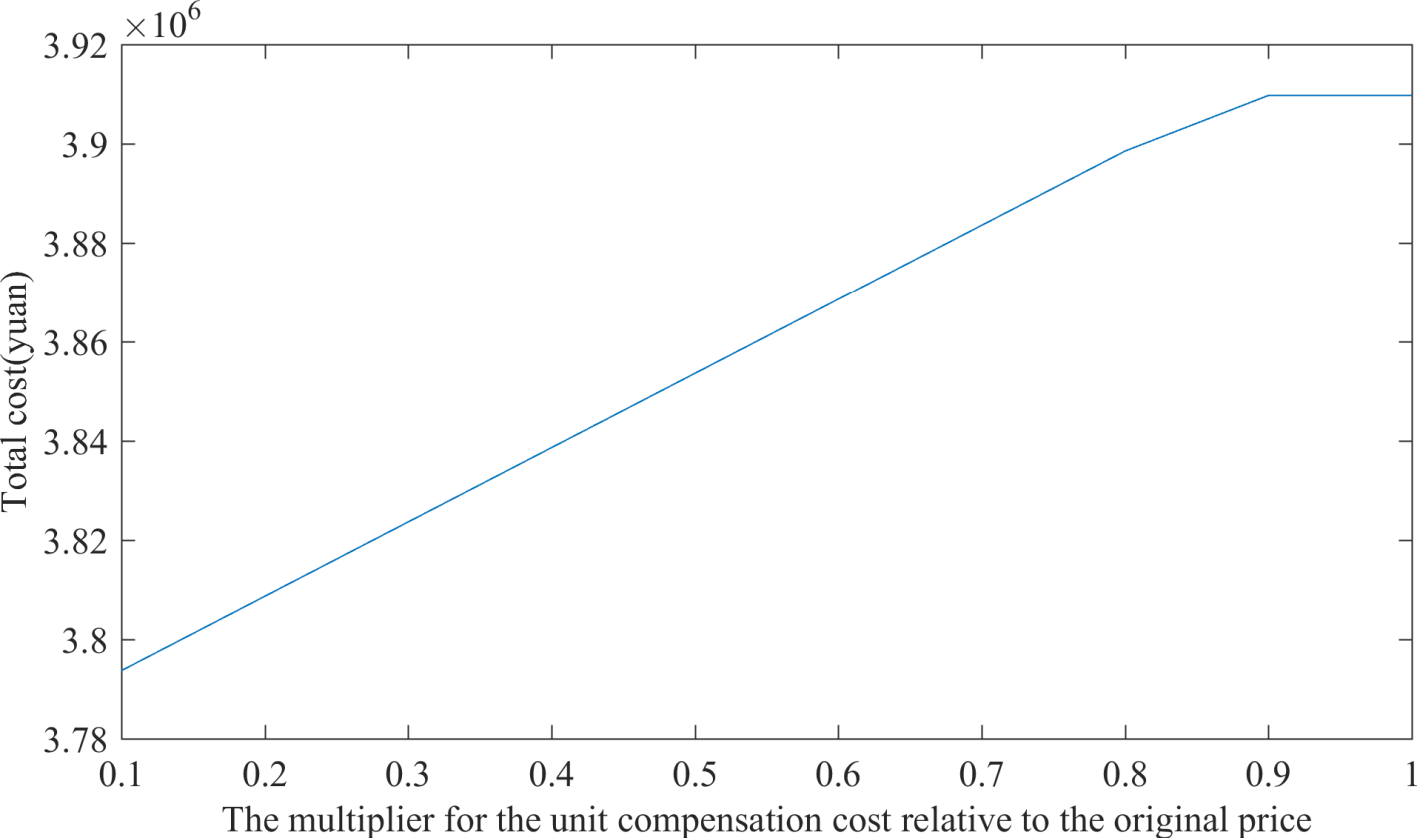
Trends in total costs with the change of unit compensation cost.

The amount of the compensation will directly affect whether the relief agencies set a higher level of commitment quantities with suppliers during the pre-disaster period or purchase more goods from the supplier at the post-disaster market price. To comprehensively analyze the impact of the level of the compensation amount on emergency rescue decisions, a sensitivity analysis for various unit compensation costs is performed. The unit compensation costs are set to the original cost multiplied by the following values: 0.1, 0.2, 0.3, 0.4, 0.5, 0.6, 0.7, 0.8, 0.9, and 1.0. For each unit compensation cost, this study analyzes the change in total cost, the quantity of materials stored at the distribution center and the supplier’s commitment quantity. The results are presented in Table 4. The relationship between unit compensation cost and total cost is illustrated in Fig 3.

**Table 4:** Total cost and material reserves for different unit compensation costs.

The multiplier for the unit compensation cost relative to the original price	Total cost (yuan)	Amount of storage at the distribution center (in thousands)	Supplier Selection and Commitment Quantities (in thousands)									
			1	2	3	4	5	6	7	9	10	11
**0.1**	3793703.22	[10,10,3]	15	115	0	400	35	360	135	10	0	149
**0.2**	3808679.22	[10,10,3]	15	115	0	400	35	360	135	10	0	149
**0.3**	3823655.22	[10,10,3]	15	115	0	400	35	360	135	10	0	149
**0.4**	3838631.22	[10,10,3]	15	115	0	400	35	360	135	10	0	149
**0.5**	3853607.22	[10,10,3]	15	115	0	400	35	360	135	10	0	149
**0.6**	3868583.22	[10,10,3]	15	115	0	400	35	360	135	10	0	149
**0.7**	3883564.70	[10,10,3]	15	115	0	400	35	360	135	0	6.5	152.5
**0.8**	3898535.22	[10,10,3]	15	115	0	400	35	360	135	10	0	149
**0.9**	3909740.58	[10,10,3]	15	30	0	245	25	242.5	22.5	10	0	77
**1.0**	3909740.58	[10,10,3]	15	30	0	245	25	242.5	22.5	10	0	77

Table 4 and Fig 3 indicate that when the unit compensation cost is in the range of 0.1 and 0.8 times the original price, the total cost will increase linearly with a gradual increase in the unit compensation cost. At this interval, there is little change in the commitment quantity of the framework agreement suppliers and the quantity of materials stored at the distribution center. This result indicates that when the unit compensation cost is less than 0.8 times the original price, it does not affect the commitment quantity decisions of the suppliers and the distribution centers. This occurs because the supplier’s market prices of goods and the material storage cost at the distribution centers are far greater than the unit compensation costs. In the range of 0.1 and 0.8 times the original price, relief agencies prefer to order more emergency relief supplies from the framework agreement providers even considering the possibility of high compensation payments; they would not store more materials at the distribution center or purchase more materials from the suppliers at market prices after the disaster occurred. When the unit compensation cost reaches 0.9 times to 1 times the original price, the storage scale of the framework agreement suppliers is significantly reduced. The amount of material that is stored at the distribution center remains unchanged, which indicates that the needs for emergency supplies can be met by ordering more materials from the suppliers at the market prices after the disaster. This result indicates that when the unit compensation cost is low, the rescue agencies will arrange for more framework agreement reserves from the supplier. When the unit compensation cost is high, the rescue agencies will purchase emergency relief supplies directly from the suppliers at the market price after the disaster. The distribution center will always store a certain amount of emergency relief supplies to meet material needs for the first distribution immediately after the disaster occurs.

Therefore, the decision-making process of the relief agencies should be concerned about the unit compensation costs of the suppliers that is specified in the framework agreement. Generally, a lower unit compensation cost will reduce the costs of the relief agencies. However, because of suppliers’ economic interests, they will not allow the rescue agencies to reduce the unit compensation costs. Therefore, at each level of unit compensation cost, a rescue organization should determine the commitment quantities of the framework agreement supplier to reduce this portion of the expenditures.

#### 4.3.3 Sensitivity analysis of post-disaster procurement expenses

To obtain a comprehensive understanding of the impact of the emergency rescue materials procurement costs on emergency rescue decision-making, this study performs a sensitivity analysis for a scenario where there are changes in the post-disaster purchase price relative to the original price to observe changes in the total cost, the quantity of materials stored at the distribution center and the commitment quantity of the framework agreement suppliers.

**Fig 4:**
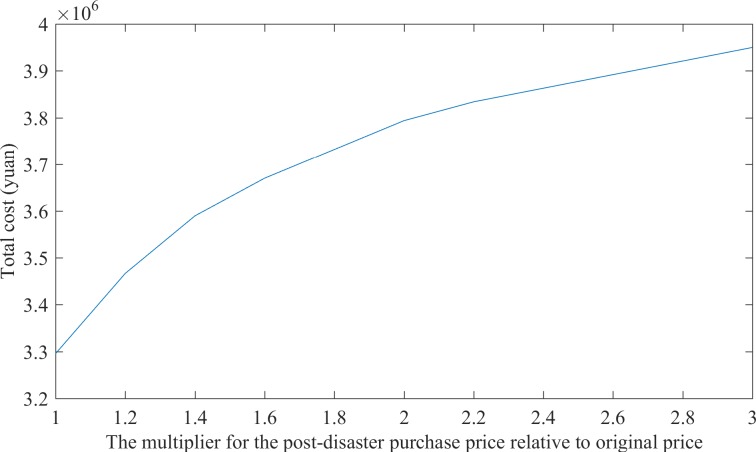
Trends of total cost as the post-disaster purchase price changes.

The multiplier for the post-disaster purchase price relative to the original price is set to 1.0, 1.2, 1.4, 1.6, 1.8, 2.0, 2.2, 2.4, 2.6, 2.8, and 3.0 when this study conducts the sensitivity analysis. The detailed data are presented in Table 5. Fig 4 illustrates the relationship between the multiplier of the post-disaster purchase price relative to the original price and the total cost.

**Table 5:** Total costs and material amount for different post-disaster purchase unit prices.

The multiplier for the post-disaster purchase price relative to original price	Total cost (yuan)	Amount of storage at the distribution center (in thousands)	Supplier Selection and Commitment Quantities (in thousands)								
			1	2	4	5	6	7	9	10	11
**1.0**	3295573.95	[10,10,3]	15	30	40	25	30	22.5	0	6.5	20
**1.2**	3466797.95	[10,10,3]	15	30	40	25	30	22.5	0	6.5	20
**1.4**	3589628.58	[10,10,3]	15	30	245	25	242.5	22.5	10	0	77
**1.6**	3670151.22	[10,10,3]	15	115	400	35	360	135	10	0	149
**1.8**	3731927.22	[10,10,3]	15	115	400	35	360	135	10	0	149
**2.0**	3793703.22	[10,10,3]	15	115	400	35	360	135	10	0	149
**2.2**	3833922.49	[10,10,3]	150	300	400	250	360	135	7.2	0	240
**2.4**	3862938.49	[10,10,3]	150	300	400	250	360	135	7.2	0	240
**2.6**	3891954.49	[10,10,3]	150	300	400	250	360	135	7.2	0	240
**2.8**	3920970.49	[10,10,3]	150	300	400	250	360	135	7.2	0	240
**3.0**	3949986.49	[10,10,3]	150	300	400	250	360	135	7.2	0	240

Table 5 and Fig 4 clearly indicate that a gradual increase in the post-disaster purchase price results in a corresponding increase in the commitment quantity of framework agreement supplier, which reduces the amount of relief supplies that are purchased from the supplier after the disaster and also reduces the corresponding emergency procurement costs. Therefore, in accordance with the specific situation of the local and surrounding areas, relief agencies predict that the price of the materials after the disaster will have greater fluctuations and will develop a large framework agreement material quantity with the suppliers before the disaster. Table 5 and Fig 4 indicate that when the multiplier of the post-disaster purchase price relative to original price is 1.4, 1.6, or 2.2, the commitment quantities of the framework agreement undergo significant changes, which implies that during these specific situations, the rescue agencies should improve the supplier's framework agreement quantity and reduce the increase of overall costs that is caused by the increase in the post-disaster procurement cost. When the post-disaster purchase price increases to a relatively high level, the change in the total cost decreases incrementally, and finally, the purchase price essentially remains unchanged. This result occurs because when the post-disaster purchase price is too high, the relief agencies would specify the sufficient amount of framework agreement materials prior to the disaster to meet the needs of disaster-stricken areas rather than purchase materials at the market price after the disaster.

In addition, Table 5 indicates that for each post-disaster purchase price, the amount of material stored by the rescue organization does not change. This result does not imply that the changes in the post-disaster purchase price do not impact the amount of emergency materials stored at the distribution center. The material storage costs at the distribution center are much higher than the framework agreement prices or the compensation prices, and the probability that a disaster will not occur is much greater than the probability that a disaster will occur. Once materials are stored at the distribution center, the cost has already been incurred. However, the occurrence of reserve costs in framework agreement suppliers is a probabilistic, and suppliers are able to share the storage costs of the relief agencies. Therefore, when the purchase price is high after the disaster, it is more reasonable to sign a larger commitment quantity with the framework agreement suppliers.

In certain areas where the supply of materials is not sufficient or the sources are relatively homogeneous, relief agencies may sign a framework agreement for a large amount of materials with suppliers, in order to prevent excessive expenses which are caused by possible fluctuations in the post-disaster material purchase price.

## 5. Conclusions

Expensive storage costs and low liquidity make it impossible to prepare for the distribution of materials in response to a large-scale disaster. Framework agreement suppliers cannot meet the most urgent needs of affected sites, which implies that they unable to alone bear the task of emergency materials preparation. Under the premise of ensuring that the most urgent needs are met in affected areas, a model that considers joint reserves between relief agencies and framework suppliers can effectively reduce the storage cost and government expenditures and optimize government emergency rescue efforts.

Using a scenario-based approach to represent demand uncertainty, we build a non-linear mathematic model to obtain suppliers’ material commitment quantities and the amount of pre-disaster material storage for the government, which indicates how cooperation between the government and the framework suppliers to store emergency materials. In addition, we propose a transportation mode ("suppliers–government distribution center—disaster area") that relieves traffic pressures in the disaster area and offers more traffic resources for emergency rescues. Furthermore, the distribution center separates the emergency relief materials into different categories that are packaged with specific ratios and directly transports the packaged goods to disaster areas. This mode greatly improves the efficiency of material distribution and reduces wastes related to relief supplies and personnel when delivering types of relief goods. Finally, we use IBM ILOG CPLEX to solve the numerical examples and verify the effectiveness of the mode. In addition, a perform sensitivity analysis of the relevant parameters is performed.

This study conducts a quantitative analysis regarding the cooperation of the government and framework suppliers to coordinate the storage of emergency rescue materials; however, this is only a preliminary exploration. In the future, scholars should conduct more systematic and specialized analyses of the topic to consider the large scale and complexity of the problem. For example, satisfaction of the affected area in regards to the emergency rescue materials depends not only on the quantity but also on the speed and time. This study does not make a quantitative analysis of satisfaction related to the timeliness of the delivery of the disaster rescue materials. These problems should be studied in greater detail.
